# Predicting prostate cancer specific-mortality with artificial intelligence-based Gleason grading

**DOI:** 10.1038/s43856-021-00005-3

**Published:** 2021-06-30

**Authors:** Ellery Wulczyn, Kunal Nagpal, Matthew Symonds, Melissa Moran, Markus Plass, Robert Reihs, Farah Nader, Fraser Tan, Yuannan Cai, Trissia Brown, Isabelle Flament-Auvigne, Mahul B. Amin, Martin C. Stumpe, Heimo Müller, Peter Regitnig, Andreas Holzinger, Greg S. Corrado, Lily H. Peng, Po-Hsuan Cameron Chen, David F. Steiner, Kurt Zatloukal, Yun Liu, Craig H. Mermel

**Affiliations:** 1 Google Health, Palo Alto, CA USA; 2 Medical University of Graz, Graz, Austria; 3 Google Health via Advanced Clinical, Deerfield, IL USA; 4 Department of Pathology and Laboratory Medicine, University of Tennessee Health Science Center, Memphis, TN USA; 5 Present Address: Tempus Labs Inc, Chicago, IL USA

**Keywords:** Prostate cancer, Prognostic markers

## Abstract

**Background:**

Gleason grading of prostate cancer is an important prognostic factor, but suffers from poor reproducibility, particularly among non-subspecialist pathologists. Although artificial intelligence (A.I.) tools have demonstrated Gleason grading on-par with expert pathologists, it remains an open question whether and to what extent A.I. grading translates to better prognostication.

**Methods:**

In this study, we developed a system to predict prostate cancer-specific mortality via A.I.-based Gleason grading and subsequently evaluated its ability to risk-stratify patients on an independent retrospective cohort of 2807 prostatectomy cases from a single European center with 5–25 years of follow-up (median: 13, interquartile range 9–17).

**Results:**

Here, we show that the A.I.’s risk scores produced a *C*-index of 0.84 (95% CI 0.80–0.87) for prostate cancer-specific mortality. Upon discretizing these risk scores into risk groups analogous to pathologist Grade Groups (GG), the A.I. has a *C*-index of 0.82 (95% CI 0.78–0.85). On the subset of cases with a GG provided in the original pathology report (*n* = 1517), the A.I.’s C-indices are 0.87 and 0.85 for continuous and discrete grading, respectively, compared to 0.79 (95% CI 0.71–0.86) for GG obtained from the reports. These represent improvements of 0.08 (95% CI 0.01–0.15) and 0.07 (95% CI 0.00–0.14), respectively.

**Conclusions:**

Our results suggest that A.I.-based Gleason grading can lead to effective risk stratification, and warrants further evaluation for improving disease management.

## Plain language summary

Gleason grading is the process by which pathologists assess the morphology of prostate tumors. The assigned Grade Group tells us about the likely clinical course of people with prostate cancer and helps doctors to make decisions on treatment. The process is complex and subjective, with frequent disagreement amongst pathologists. In this study, we develop and evaluate an approach to Gleason grading based on artificial intelligence, rather than pathologists’ assessment, to predict risk of dying of prostate cancer. Looking back at tumors and data from 2,807 people diagnosed with prostate cancer, we find that our approach is better at predicting outcomes compared to grading by pathologists alone. These findings suggest that artificial intelligence might help doctors to accurately determine the probable clinical course of people with prostate cancer, which, in turn, will guide treatment.

## Abstract (web summary)

Wulczyn et al. utilise a deep learning-based Gleason grading model to predict prostate cancer-specific mortality in a retrospective cohort of radical prostatectomy patients. Their model enables improved risk stratification compared to pathologists’ grading and demonstrates the potential for computational pathology in the management of prostate cancer.

## Introduction

Prostate cancer affects one in nine men in their lifetime^1^, but disease aggressiveness and prognosis can vary substantially among individuals. The histological growth patterns of the tumor, as characterized by the Gleason grading system, are a major determinant of disease progression and criterion for selection of therapy. Based on the prevalence of these patterns, one of five Grade Groups (GG) is assigned^2^. The GG is among the most important prognostic factors for prostate cancer patients, and is used to help select the treatment plan most appropriate for a patient’s risk of disease progression^3^.

The Gleason system is used at distinct points in the clinical management of prostate cancer. For patients undergoing diagnostic biopsies, if tumor is identified, the GG impacts the decision between active surveillance vs. definitive treatment options, such as surgical removal of the prostate or radiation therapy^3^. For patients who subsequently undergo a surgical resection of the prostate (radical prostatectomy), the GG is one key component of decisions regarding adjuvant treatment, such as radiotherapy or hormone therapy^4,5^. In large clinical trials, use of adjuvant therapy following prostatectomy has demonstrated benefits, such as improved progression-free survival for some patients, but can also result in substantial adverse side effects^6–8^. As such, several post-prostatectomy nomograms^9^ have been developed, in order to better predict clinical outcomes following the definitive treatment, with the goal of identifying the patients most likely to benefit from adjuvant therapy. Gleason grading of prostatectomy specimens represents a key prognostic element in many of these nomograms, and is a central component of the risk categories defined by the National Comprehensive Cancer Network^5^.

Due to the complexity and intrinsic subjectivity of the system, Gleason grading suffers from large discordance rates between pathologists (30–50%)^10–15^. However, grades from experts (such as those with several years of experience, primarily practicing urologic pathology, or those with urologic subspeciality training) are more consistent and result in more accurate risk stratification than grades from less experienced pathologists^16–19^, suggesting an opportunity to improve the clinical utility of the system by improving grading consistency and accuracy. To this end, several artificial intelligence (A.I.) algorithms for Gleason grading have been developed and validated, using expert-provided Gleason scores^20–23^. However, an evaluation of the prognostic value of these algorithms and a direct comparison to the prognostic value of Gleason grading provided by pathologists has not been conducted. While the GG for biopsies, as well as prostatectomy specimens both provide important prognostic information^2^, retrospective studies to evaluate long-term clinical outcomes is more straightforward from prostatectomy cases given widely divergent treatment pathways following biopsy alone.

Building on prior work^22,24^, we first trained an A.I. system to accurately classify and quantitate Gleason patterns on prostatectomy specimens, and further demonstrate that A.I.-based Gleason pattern (GP) quantitations can be used to provide better risk stratification than the Gleason GG from the original prostatectomy pathology reports.

## Methods

### Data

All available slides for archived prostate cancer resection cases between 1995 and 2014 in the Biobank Graz^25,26^ at the Medical University of Graz were retrieved, de-identified, and scanned using a Leica Aperio AT2 scanner at 40× magnification (0.25 μm/pixel). The standard protocol for radical prostatectomy submission at the institution was to submit the entire prostate (right and left lobes, additionally divided into ventral and dorsal portions, and serially sectioned apex to base approximately every 3–5 mm). To our knowledge, there was no change in surgical procedure type over the time period studied. Robotic surgery was not used.

Gleason patterns (Gleason scores) were extracted from the original pathology reports and translated to their corresponding GG^2^. Tertiary patterns, which were reported in only 22 of the 2807 cases (<1%), were not used in this study. Clinicopathologic variables, such as pathologic TNM staging, were also extracted from the pathology reports. Disease-specific survival (DSS) was inferred from International Classification of Diseases codes obtained from medical death certificates from the Statistik Austria database. Codes considered for prostate cancer-related death were C61 (malignant neoplasm of prostate) and C68 (malignant neoplasm of other and unspecified urinary organs). Institutional Review Board approval for this retrospective study, using anonymized slides and associated pathologic and clinical data, was obtained from the Medical University of Graz (Protocol no. 32-026 ex 19/20). Need for informed consent was waived because the project was performed with anonymized data.

Validation set 1 included all available cases from 1995 to 2014 after application of the exclusion criteria (*n* = 2807; Table 1 and Supplementary Fig. S1). Because Gleason scoring at the Medical University of Graz was adopted in routine practice from 2000 onward, validation set 2 included all cases from 2000 onward for which a Gleason score was available (*n* = 1517; Table 1). Sensitivity analysis for inclusion of Gleason grades prior to the year 2000 (before Gleason scoring became routine at the institution) is presented in Supplementary Table S1. The specific purpose of validation set 2 is to allow for a direct comparison of the prognostic performance of the A.I. with that of the pathologist Gleason Grades.

**Table 1:** Cohort characteristics.

		Validation set 1	Validation set 2 (subset of set 1)
Number of cases		2807	1517
Number of slides	Total	83,645	47,626
	Median per case (interquartile range)	29 (25, 34)	30 (26, 35)
Overall survival (OS)	Median years of follow-up (interquartile range)	13.1 (8.5, 17.2)	11.2 (7.4, 15.2)
	Censored (%)	2150 (77%)	1306 (86%)
	Observed (%)	657 (23%)	211 (14%)
Disease-specific survival (DSS) (%)	Censored	2673 (95%)	1464 (97%)
	Observed	134 (5%)	53 (3%)
Grade Group (%)	1	611 (22%)	608 (40%)
	2	476 (17%)	473 (31%)
	3	224 (8%)	224 (15%)
	4	128 (5%)	127 (8%)
	5	85 (3%)	85 (6%)
	Unknown	1283 (46%)	0 (0%)
Pathologic T-stage (%)	T2	1640 (58%)	1113 (73%)
	T3	791 (28%)	366 (24%)
	T4	25 (1%)	6 (<1%)
	Unknown	351 (13%)	32 (2%)
Age at diagnosis (%)	<60	952 (34%)	537 (35%)
	60–70	1546 (55%)	817 (54%)
	≥70	309 (11%)	163 (11%)
Margin status (%)	Negative	448 (16%)	153 (10%)
	Positive	242 (9%)	96 (6%)
	Unknown	2117 (75%)	1268 (84%)
Pathologic N-stage (%)	N0	1395 (50%)	879 (58%)
	N1	77 (3%)	62 (4%)
	N2	13 (<1%)	4 (<1%)
	N3	10 (<1%)	8 (1%)
	Unknown	1312 (47%)	564 (37%)
Received hormone or chemotherapy (%)	Yes	53 (2%)	33 (2%)
	No/unknown	2754 (98%)	1484 (98%)
Received radiation therapy (%)	Yes	277 (10%)	176 (12%)
	No/unknown	2530 (90%)	1341 (88%)
Biochemical recurrence (%)	Censored	338 (12%)	228 (15%)
	Observed	95 (3%)	55 (4%)
	No follow-up	2374 (85%)	1234 (81%)

Validation set 1 contains all prostatectomy cases from the Biobank Graz between 1995 and 2014. Validation set 2 was derived by first considering cases in the Gleason grading era at the institution (years 2000–2014; *n* = 2191), and then further filtering for cases where a Gleason score was recorded and available in the pathology report (*n* = 1517).

All slides underwent manual review by pathologists (see “Pathologist cohort and QC details” in the Supplementary Methods) to confirm stain type and tissue type. Inclusion/exclusion criteria are described in Supplementary Fig. S1. Briefly, immunohistochemically stained slides were excluded from analysis and only slides containing primarily prostatic tissue were included. Slides containing exclusively prostatic tissue were included in their entirety. Slides with both prostatic tissue and seminal vesicle tissue were included, but processed using a prostatic tissue model meant to provide only prostatic tissue to the Gleason grading model (for more details on its development and performance, see “Prostatic tissue segmentation model” in Supplementary Methods and Supplementary Figs. S1 and S2).

### Gleason grading model

We previously developed two A.I. systems: one for Gleason grading prostatectomy specimens^24^ based on a classic “inception” neural network architecture, and a second for Gleason grading biopsy specimens based on a customized neural network architecture^22^. For this work, we used the prostatectomy dataset from the first study to train a new model using the customized neural network architecture introduced in the second study. The training dataset contained 112 million pathologist-annotated “image patches” from an independent set of prostatectomy cases from different institutions than the validation data used in this study. Briefly, the system takes as input 512 × 512 pixel image patches (at 10× magnification, 1 μm per pixel) and classifies each patch as one of four categories: nontumor, GP 3, 4, or 5. The hyperparameters used for training this network were determined using a random grid search that optimized for tuning set classification accuracy over 50 potential settings, and are described in Supplementary Table S2 and “Gleason grading model tuning” in the Supplementary Methods.

### A.I. risk scores and risk groups

The Gleason grading model was run at stride 256 (at 10× magnification, 1 μm per pixel) on all prostate tissue patches. The classification of each patch as nontumor or GP 3, 4, or 5 was determined via argmax on re-weighted predicted class probabilities^24^. For each case, the percentage of prostate tumor patches that belong to Gleason patterns 3, 4, and 5 were subsequently computed by counting the numbers of patches categorized as each pattern across all slides for each case. A.I. risk scores were computed by fitting a Cox regression model using these case-level GP percentages as input, and the right-censored outcomes as the events (see workflow diagram in Supplementary Fig. S2). This approach was pursued first (rather than direct mapping of %GPs to GG as done by pathologists) due to the prognostic importance of precise GP quantitation^27^, as well as the exhaustive nature of A.I. grading that rarely leads to classifications of GG1 (e.g., 100% GP3) and GG4 (e.g., 100% GP4). Sensitivity analyses evaluating additional ways of obtaining risk groups from %GPs, including direct mapping of %GPs to GG and a temporal-split methodology, demonstrated qualitatively similar results and are presented in Supplementary Table S3.

GP 3 percentage was dropped as an input feature to avoid linear dependence between features. Leave-one-case-out cross-validation was used to adjust for optimism, similar to the tenfold cross-validation used in Epstein et al.^2^. A.I. risk groups were derived from the A.I. risk scores by discretizing the A.I. risk scores to match the number and frequency of pathologist GG in validation set 2. Discretization thresholds for both validation sets are provided in Supplementary Table S4.

### Statistical analysis

Primary and secondary analyses were prespecified and documented prior to evaluation on the validation sets. The primary analysis consisted of the comparison of *C*-indices for DSS between pathologist GG and the A.I. risk scores (Table 2). The secondary analysis consisted of the comparison between *C*-indices for pathologist GG and the discretized A.I. risk groups. All other analyses were exploratory.

**Table 2:** *C*-index for pathologist and A.I. grading.

	*C*-index [95% CI]	
	Validation set 1 (*n* = 2807 cases)	Validation set (*n* = 1517 cases)
(A) Pathologist Grade Groups	N/A^a^	0.79 [0.71, 0.86]
(B) A.I. risk score (continuous)	**0.84 [0.80–0.87]**	**0.87 [0.81, 0.91]**
(C) A.I. risk groups (discretized)	0.82 [0.78–0.85]	0.85 [0.79, 0.90]
(D) Average of (A) and (C)	N/A^a^	0.86 [0.80–0.91]

The A.I. risk score (B) is a continuous risk score from a Cox regression fit on Gleason pattern percentages from the A.I. The A.I. risk group (C) is a discretized version of the A.I. risk score. The discretization was done to match the number and frequency of pathologist Grade Groups in validation set 2. (D) Represents the average of the Pathologist Grade Group and A.I. risk groups. In validation set 2, the *C*-index for the A.I. risk score was statistically significantly higher than that for the pathologists’ Grade Group (*p* < 0.05, prespecified analysis). Bold indicates the highest value in each column (dataset).

^a^Not available because pathologist Grade Groups were not available for all cases in validation set 1 due to the earlier time period.

The prognostic performance of the pathologist GG, the A.I. risk scores, and the A.I. risk groups were measured using Harrel’s *C*-index^28^, a generalization of area under the receiver operating characteristic curve for time-censored data. Confidence intervals for both the *C*-index of A.I. and pathologists, and the differences between them, were computed via bootstrap resampling^29^ with 1000 samples.

In Kaplan–Meier analysis of the pathologist GG and A.I. risk groups, the multivariate log-rank test was used to test for differences in survival curves across groups. All survival analysis were conducted using the Lifelines python package^30^ (version 0.25.4).

### Reporting summary

Further information on research design is available in the Nature Research Reporting Summary linked to this article.

## Results

### Summary of cohort

All archived slides in prostatectomy cases from 1995 to 2014 at the Biobank at the Medical University of Graz in Austria^25,26^ were digitized. After excluding nine cases for death within 30 days of surgery and eight cases without evidence of prostate cancer in the resection, 2807 cases remained (Supplementary Fig. S1). The median follow-up time was 13.1 years (interquartile range 8.5–17.2). These cases were grouped into two validations sets: all cases (validation set 1) and the subset of cases from 2000 to 2014 for which Gleason grading was performed at the time of pathologic diagnosis and provided in the final pathology report (*n* = 1,517 cases, validation set 2). Descriptive statistics for both validation sets are provided in Table 1.

### A.I. and pathologist prognostication

For each case, an A.I. algorithm assessed the tumor composition and output percentages for the three different Gleason patterns (%GP3, %GP4, and %GP5). We fit a Cox proportional hazards regression model directly on these percentages to produce continuous A.I. risk scores (Supplementary Table S4), using leave-one-out cross-validation to “adjust for optimism”^2^. On validation set 1, this continuous A.I. risk score achieved a *C*-index of 0.84 (95% CI 0.80–0.87; Table 2). In prespecified primary analysis, on validation set 2, the *C*-index for the A.I. risk score (0.87) was significantly greater than the *C*-index for the GG obtained from the original pathology report (0.79), an improvement of 0.08 (95% CI 0.01–0.15).

**Fig. 1:**
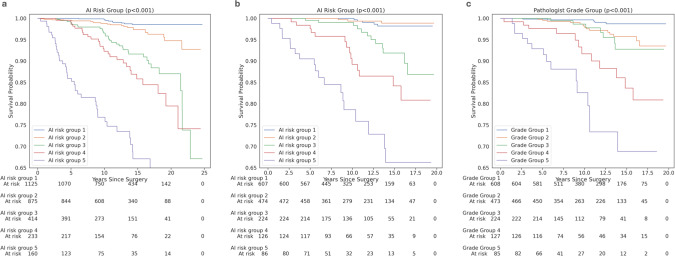
Kaplan–Meier curves for A.I. and pathologist. Kaplan–Meier (KM) curves for **a** A.I. risk groups on validation set 1, **b** A.I. risk groups on validation set 2, and **c** pathologist Grade Groups on validation set 2. The colored lines represent the risk groups categorized by the A.I. or pathologist: 1 in blue; 2 in orange; 3 in green; 4 in red; and 5 in purple. *P* values were calculated using the log-rank test.

To provide an additional comparison to pathologists’ GG categorizations, we discretized the A.I. risk scores into five “A.I. risk groups” such that the number of cases per risk group matched the number of cases in the corresponding GG. Similar to the A.I. risk score, the *C*-index for the A.I. risk groups (0.85) was also greater than the *C*-index for the pathologist GG (Table 2), an improvement of 0.07 (95% CI 0.00–0.14). Furthermore, Kaplan–Meier analyses showed significant risk stratification across A.I. risk groups across both validation sets (*p* < 0.001 for log-rank test, Fig. 1) and univariable Cox regression analyses showed higher hazard ratios for higher A.I. risk groups (Supplementary Table S5).

### Controlling for treatment

To explore the extent to which post-surgery treatment impacted the prognostication from the GG at prostatectomy, we conducted additional subset analyses on cases with and without known adjuvant or salvage therapy from the institution, where the prostatectomy was conducted, the Medical University of Graz (Supplementary Fig. S3). For validation set 2, on the subset of cases without known additional treatment (*n* = 1327) the *C*-index for the A.I. risk score (0.85) remained greater than the *C*-index for the pathologist GG (0.77), an improvement of 0.08 (95% CI 0.01–0.17). On the subset of cases with known additional treatment (*n* = 190), similarly the *C*-index for the A.I. risk score (0.88) compared favorably to the *C*-index of the pathologist GG (0.79), delta of 0.09 (95% CI −0.03 to 0.24). Similar results were observed for validation set 1 (Supplementary Fig. S3).

### Controlling for other features

We also evaluated the prognostic performance of the A.I. in the context of additional important pathologic features. Kaplan–Meier analyses showed significant risk stratification across A.I. risk groups even within groups defined by low and high T-category (*p* < 0.001 for log-rank test, Supplementary Fig. S4). Furthermore, using the A.I. risk groups in a multivariable Cox model that also included T-category, surgical margins, and lymph node metastasis status gave a *C*-index that trended higher than using the pathology report-derived GG, and A.I. risk scores remained independently prognostic with respect to these additional features (Supplementary Tables S6–8).

### Substratification of pathologist grade groups

**Fig. 2:**
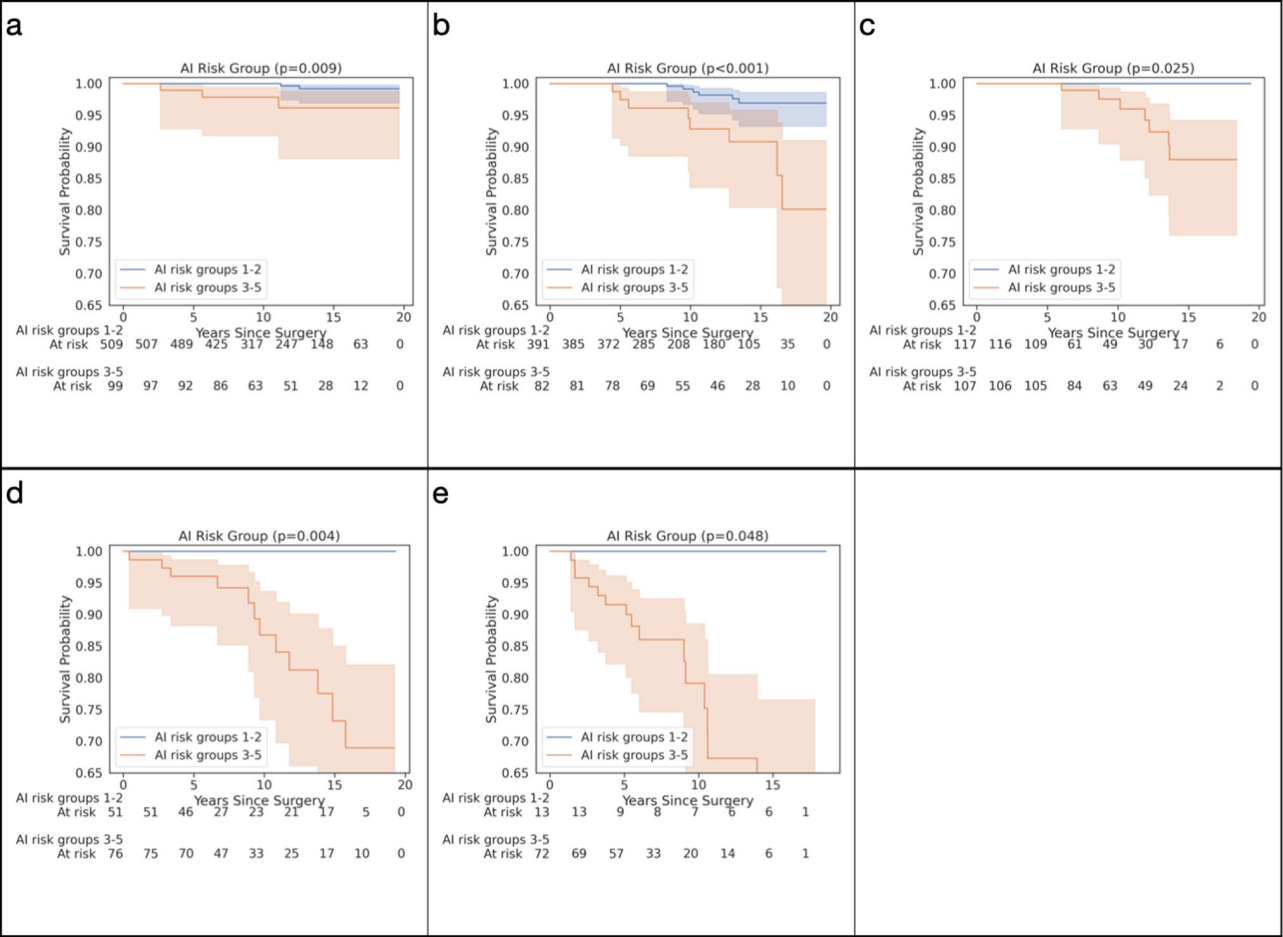
Substratification of patients by A.I. as risk groups 1–2 vs. 3–5 within each pathologist-determined GG. A.I. risk groups 1 and 2 are represented in blue, whereas A.I. risk groups 3–5 are represented in orange. Substratification of pathologist-determined **a** GG1, **b** GG2, **c** GG3, **d** GG4, and **e** GG5. Shaded areas represent 95% confidence intervals.

To better understand discordances between the A.I. risk groups and pathologist GG, we first compared 10-year DSS rates for cases, where the A.I. risk group was higher or lower than the pathologist GG (Supplementary Table S9). Within each pathologist-determined GG, the 10-year survival rates were higher for cases, where the A.I. provided a lower risk classification, especially for GG ≥ 3. The survival rates also tended to be lower, where the A.I. provided a higher-risk classification. Second, risk stratification by the A.I.’s risk groups 1–2 vs. 3–5 remained significant within each pathologist-determined GG (Fig. 2). In particular, among patients with pathologist GG 3–5, a sizable subgroup (181 of 436, 42%) were assigned A.I. risk groups of 1–2, and these patients did not experience any disease-specific mortality events (Supplementary Table S9 and Fig. 2).

### Exploratory analysis: combining A.I. and pathologists grades

We further explored the potential benefit of combining the A.I. system and pathologist grading by evaluating a simple “ensembling” approach. The arithmetic mean of the A.I. risk group and pathologist-provided GG resulted in a *C*-index of 0.86 (95% CI 0.80–91) compared to 0.85 for the A.I. risk groups alone (Table 2). This small improvement was not statistically significant. Furthermore, qualitative analysis of algorithm and pathologist discordances suggests several ways, in which the algorithmic grading and pathologist grading may be complementary, including consistent grading of regions by the A.I. which may be variably overgraded by pathologists, or identification of small, high grade regions that may otherwise be missed by pathologists.

### Exploratory analysis: intra- and inter-scanner variability

Finally, we conducted intra-scanner and inter-scanner variability analysis across four scanner types, finding that intra-scanner *R*^2^ consistently exceeded 0.99, whereas inter-scanner *R*^2^ varied more, but was still >0.94 (Supplementary Table S10).

## Discussion

In this study, we have validated the ability of a Gleason grading A.I. system to risk-stratify patients using an independent dataset of over 2800 prostatectomy cases, with a median of 13 years of follow-up. The A.I. system demonstrated highly effective risk stratification and, in prespecified primary analysis, provided significantly better risk stratification than GGs obtained from the original pathology reports.

After prostatectomy, adjuvant radiotherapy for patients with high-risk pathological features has been shown to reduce rates of disease recurrence in multiple clinical trials^6–8^, and to improve overall survival in some cohorts^31^. Given their prognostic value, Gleason grades represent a key factor in adjuvant therapy decisions, with NCCN practice guidelines suggesting higher-risk patients be considered for adjuvant therapy^3^. However, use of adjuvant radiotherapy can cause adverse effects, contributing to low utilization of this treatment option^32^ despite there being a subset of patients who would likely benefit. While risk stratification tools, such as nomograms (in which the Gleason score is among the most prognostic factors)^9^ and molecular tests^33^, have been developed, selection of patients for adjuvant therapy post-prostatectomy remains a difficult task^3^. Given the ability of the A.I. to provide significant risk stratification among patients most likely to consider adjuvant therapy (GG 3–5 and pT3 and above, Supplementary Fig. S4B), our results suggest that the A.I. risk score could be particularly useful for informing adjuvant therapy decisions. Evaluation of whether additional prognostic value can be obtained by combining the A.I. risk score with existing prognostic tools, such as nomograms and molecular approaches, is also warranted.

The A.I. system may also contribute to clinical decision making by directly assisting pathologist grading as a computer-aided diagnostic (CADx) tool. Prior work has shown that a CADx tool for Gleason grading can improve grading consistency and accuracy by pathologists, with pathologists benefiting from the consistent grading provided by the A.I., while also correcting and overriding unexpected A.I. errors as needed^34,35^. Given the prognostic importance of expertise in pathology review^19^, and the scarcity of specialty pathologists in low-income and middle-income countries^36^, utilization of the A.I. system as an assistive tool during prostatectomy review has the potential to improve access to consistent, accurate grading, and may ultimately result in grading that more accurately predicts patient outcome.

While not directly comparable due to differences in cohorts and study design, the prognostic performance observed for the pathologist Gleason grading in this cohort is largely consistent with prior work evaluating associations of pathologist grading and clinical outcomes (*C*-indices of 0.70–0.83 for GG and biochemical recurrence^2,37,38^, and 0.80 for the recent STAR-CAP clinical prognostic grouping and DSS^39^). Interestingly, the univariate hazard ratios for %GP4 and %GP5 were comparable (1.48 and 1.51 for each 10% increase in the respective pattern). These findings are consistent with Sauter et al., who found the presence of any %GP5 had strong adverse prognostic implications on Gleason score 7 patients, but additional increases of the %GP5 had reduced further impact on prognosis^40^.

Several other works have developed Gleason grading algorithms, though without validating them on clinical outcomes^20,21,23^. In addition, Yamamoto et al. recently demonstrated the ability to directly learn prognostic histologic features in prostate cancer specimens that correlate with patient outcomes^41^. The present study complements prior work by building upon an extensively validated Gleason system to provide A.I. risk assessments that are directly interpretable by pathologists, and utilizing a large independent dataset with long-term clinical follow-up for direct validation of these assessments on patient outcomes.

This study has some limitations. First, the Gleason grading system has evolved over the time period, in which data was collected for this study, including changes to the reporting of minor Gleason patterns, potentially contributing to inconsistencies in grading between pathologists and underestimating the prognostic performance of the GG in the original report. Relatedly, we did not have access to the raw GP percentages used by pathologists to determine the GG, which limited comparison with continuous pathologist risk scores. Similarly, the A.I. and pathologist grading differ in that A.I. grading does not grade tumor within seminal vesicle regions, nor does it take into account concepts, such as dominant or codominant nodules, but instead evaluates the entire case holistically. Next, this study focuses on prostatectomy specimens. The benefit of prostatectomy-based analysis is that the interpretation of prognostication performance in resections is more straightforward than for biopsies due to less divergent postoperative treatment pathways^42^. Additional research to compare the prognostic value of A.I.-based Gleason scoring to that of subspecialist pathologists or consensus panels can help further contextualize the A.I.’s performance. Future work to validate an accurate A.I. system’s prognostic utility on biopsies may provide additional opportunities to inform and improve post-biopsy clinical decisions. In addition to Gleason grading, pathologists review cases for additional criteria, including TNM staging, cancer variants^43^, and other pathologic findings not evaluated by our system. Therefore, the potential benefits of integrating our A.I. system into a routine pathology workflow will ultimately need to be evaluated in prospective studies. Finally, although this work was done on a dataset from a different institution than the datasets used to develop the A.I., additional validation on diverse cohorts will be required to further validate these findings.

To conclude, we have validated the ability of an A.I. Gleason grading system to effectively risk-stratify patients on a large retrospective cohort, outperforming the Gleason GG in the original report. We look forward to future research involving the clinical integration and evaluation of the impact of A.I. for improving patient care.

## Supplementary information

Supplementary Information

Reporting Summary

## Data Availability

This study analyzed datasets containing archived anonymized pathology slides, clinicopathologic variables, and outcomes information from the Institute of Pathology and the Biobank at the Medical University of Graz. The datasets are not publicly available to respect patient privacy, and interested researchers should contact K.Z. (kurt.zatloukal@medunigraz.at) to inquire about access; requests for noncommercial academic use will be considered and require ethics review.

## Code availability

The deep learning framework (TensorFlow) used in this study is available at https://www.tensorflow.org. The deep learning architecture for the Gleason grading model is detailed in prior work^22^. All survival analyses were standard and conducted using Lifelines^30^, an open-source Python library. The trained model has not yet undergone regulatory review and cannot be made available at this time. Interested researchers can contact C.M. (cmermel@google.com) for questions on its status and access.
