# Supplementary material for: Predicting prostate cancer specific-mortality with artificial intelligence-based Gleason grading
Source: Commun Med (Lond). 2021 Jun 30;1:10. doi: 10.1038/s43856-021-00005-3 (PMC9053226; doi:10.1038/s43856-021-00005-3)
Supplement: Supplementary file 1 — Supplementary Information [file 43856_2021_5_MOESM1_ESM.pdf]

Supplementary Information for: Predicting prostate cancer specific-mortality with artificial intelligence-based Gleason grading

Authors:
Ellery Wulczyn[1,†], Kunal Nagpal[1,†], Matthew Symonds[1], Melissa Moran[1], Markus Plass[2], Robert Reihs[2], Farah Nader[2], Fraser Tan[1], Yuannan Cai[1], Trissia Brown[3], Isabelle Flament-Auvigne[3], Mahul B. Amin[4], Martin C. Stumpe[5], Heimo Müller[2], Peter Regitnig[2], Andreas Holzinger[2], Greg S. Corrado[1], Lily H. Peng[1], Po-Hsuan Cameron Chen[1], David F. Steiner[1], Kurt Zatloukal[2], Yun Liu[1,‡], Craig H. Mermel[1,‡]

Affiliations:
[1]Google Health, Palo Alto, CA, USA
[2]Medical University of Graz, Graz, Austria
[3]Work done at Google Health via Advanced Clinical, Deerfield, IL, USA
[4]Department of Pathology and Laboratory Medicine, University of Tennessee Health Science Center, Memphis, TN, USA
[5]Work done at Google Health. Present address: Tempus Labs Inc, Chicago, IL, USA
[†]These authors contributed equally
[‡]These authors jointly supervised the work

Address correspondence to: Y.L. (liuyun@google.com), K.Z. (kurt.zatloukal@medunigraz.at)

Supplementary Methods

Prostatic Tissue Segmentation Model

In order to collect data for model development, pathologists were asked to coarsely outline extraprostatic tissue and seminal vesicle regions across 221 slides from The Cancer Genome Atlas[1] and previously-digitized de-identified slides from the Naval Medical Center San Diego[2]. Extraprostatic tissue and seminal vesicle annotations were combined into a single 'Extraprostatic Tissue' class. An additional 150 de-identified slides were randomly sampled from the Gleason grading dataset (see "Gleason Grading Model" in the Methods), and any benign or Gleason pattern 3, 4, or 5 annotation was considered to be part of the 'Prostatic Tissue' class.

The resulting 371 slides were randomly split into a training and tuning split. A convolutional neural network, using the same architecture, training methodology, and hyperparameter tuning methodology described for the Gleason Grading model, was trained for the binary 'Extraprostatic Tissue' vs. 'Prostatic Tissue' task, with a resulting AUC of 0.99 across 64,000 image patches sub-sampled from the tuning set with a 1:1 class ratio. The threshold for binarization was chosen to achieve 97% precision (at 84% recall) of prostatic tissue.

Gleason Grading Model Tuning

Model tuning was conducted by sub-sampling 64,000 image patches from the region-annotated tuning set with a class ratio of 2:1:1:1 for classes Benign:GP3:GP4:GP5. Compared to the Gleason grading network used in the prior study[2], the tuning set concordance with the majority-vote of 3 pathologists in classifying individual regions of the new network improved from 0.76 to 0.78 on this sub-sampled set.

Pathologist Cohort and QC Details

Manual pathologist reviews for slides' stain and tissue types were performed by a cohort of 19 US board-certified pathologists across 11 states and 2 non-US trained pathologists. The median of years of experience amongst this cohort was 11 (range: 2-25). When reviewing the stain type, pathologists were asked to classify each slide as H&E, Unstained, IHC, or Unable to evaluate. When reviewing the tissue type of each slide, pathologists were asked to select all of the following that apply for tissue present on the slide: Prostate, Seminal vesicle, Colon, Lymph node, Unable to determine, or Other.

Supplementary Tables

Supplementary Table S1. Sensitivity analysis for years included in validation set 2
All results represent C-index with 95% confidence intervals in square braces. The middle column presents the original validation set 2 analysis comprising all cases with a Gleason score provided in the original pathology report from 2000 onwards (at which time Gleason scoring became common practice at the institution); the rightmost column presents analysis comprising all cases with a Gleason score available (including those before 2000). The primary analysis was conducted on cases from 2000 onwards to improve cohort standardization, while sensitivity analysis was conducted for completeness to additionally consider the small number of cases before 2000 for which Gleason scores were available.

**Year of analysis**	**2000-2014**	**1995-2014**
No. of cases	1,517	1,524
Pathologist Grade Groups	0.79 [0.71, 0.86]	0.78 [0.71, 0.85]
A.I. risk score	0.87 [0.81, 0.91]	0.86 [0.81, 0.91]
A.I. risk group	0.85 [0.79, 0.90]	0.85 [0.80, 0.90]

Supplementary Table S2. Hyperparameters for training Gleason grading model.

	**Gleason Grading Model**	**Prostatic Tumor Segmentation**
Architecture	Custom TuNAS Architecture[3]<br>L2 Weight Decay: 0.004	
Color perturbations	Saturation delta: 0.80<br>Brightness delta: 0.96<br>Contrast delta: 0.17<br>Hue delta: 0.02	
Learning rate schedule	Exponential decay schedule<br>Base rate: 0.0042<br>Decay rate: 0.95<br>Decay steps: 51,733 steps	Exponential decay schedule<br>Base rate: 0.0001<br>Decay rate: 0.90<br>Decay steps: 25,000 steps
RMSProp optimizer	Decay: 0.95<br>Momentum: 0.7<br>Epsilon: 0.001	Decay: 0.95<br>Momentum: 0.7<br>Epsilon: 0.001
Other	Image input magnification: 10X (1 µm/pixel)<br>Loss function: softmax cross-entropy<br>Batch size: 32	Image input magnification: 5X (2 µm/pixel)<br>Loss function: softmax cross-entropy<br>Batch size: 16

Supplementary Table S3. Sensitivity analysis evaluating different ways of obtaining discrete A.I. risk groups from A.I. Gleason pattern percentages.
(1) Risk scores from a Cox regression model fit on A.I. Gleason pattern percentages were generated via leave-one-out cross-validation (LOOCV). These risk scores were discretized to (1.1) match the pathologist Grade Group distribution in validation set 2 or (1.2) match the pathologist Grade Group distribution in the cohort described in Epstein et al.. (2) Cases from 1995-1999 from validation set 1 were used to train a Cox regression model on A.I. Gleason pattern percentages. Predicted risk scores from this model on validation set 2 cases (2000-2014) were discretized to (2.1) match the pathologist Grade Group distribution in validation set 2 or (2.2) match the pathologist Grade Group distribution in the cohort described in Epstein et al.. (3) A.I. Gleason pattern percentages were mapped to discrete risk groups using a similar rule-based mapping used by pathologists to determine the Grade Group from Gleason pattern percentages[4].

	C-index [95%CI]	
Method	Validation Set 1	Validation Set 2
1) LOOCV		
1.1) Validation set 2 GG distribution	0.82 [0.78, 0.85]	0.85 [0.79, 0.90]
1.2) Epstein et. al. GG distribution	0.82 [0.78, 0.85]	0.85 [0.80, 0.90]
2) Temporal split		
2.1) Validation set 2 GG distribution	N/A	0.86 [0.80, 0.90]
2.2) Epstein et. al. GG distribution	N/A	0.86 [0.80, 0.90]
3) Rule-based	0.80 [0.75, 0.84]	0.84 [0.78, 0.88]

Supplementary Table S4. A.I. risk score thresholds for discretizing into risk groups.
The A.I. risk score is the output of a cox regression model of the form: exp [log(HR_{GP4}) * (10 * %GP4 - $\underline{\%GP4}$) + log(HR_{GP5}) * (10 * %GP5 - $\underline{\%GP5}$)], where %GP4 and %GP5 are fractions in the range [0, 1], and the multiplier of 10 scales the variable such that the hazard ratios are per 10% increase in the associated patterns. $\underline{\%GP4}$ and $\underline{\%GP5}$ are normalization constants. For the primary analysis, A.I. risk scores were computed via leave-one-out cross validation (LOOCV), meaning that there is actually a separate cox regression model for each case. To provide concrete examples, the model fit on all validation set 1 cases is exp [log(1.48) * (10 * %GP4 - 2.35) + log(1.51) * (10 * %GP5 - 0.94)]. The model fit on all validation set 2 cases is exp [log(1.58) * (10 * %GP4 - 2.22) + log(1.63) * (10 * %GP5 - 0.85)].

**A.I. Risk Group**	**Lower threshold for risk group**	
	**Validation set 1**	**Validation set 2**
GG2	0.53	0.48
GG3	1.64	1.70
GG4	4.53	4.91
GG5	9.58	14.24

Supplementary Table S5. Hazard ratios for A.I. Gleason pattern percentages Hazard ratios from Cox regression analysis on A.I. Gleason pattern percentages. Gleason pattern percentages from pathologists were not available from the clinical reports for these cohorts**.** Hazard ratios represent the risk increase per 10 percentage point increase in the respective pattern. P-values were computed from a Wald test.

	**Validation Set 1**		**Validation Set 2**	
	Hazard ratio [95%CI]	P-value	Hazard ratio [95%CI]	P-value
**A.I. Gleason pattern percentage**				
% GP3	1.0 (reference)	-	1.0 (reference)	-
% GP4	1.48 [1.37, 1.60]	<0.001	1.58 [1.39, 1.79]	<0.001
%GP5	1.51 [1.41, 1.61]	<0.001	1.63 [1.46, 1.82]	<0.001

Supplementary Table S6. Hazard ratios for pathologist Grade Group and A.I. risk group
Hazard ratios from univariable Cox regression models for pathologist Grade Group and A.I. risk groups. P-values were computed from a Wald test.

	Validation Set 1		Validation Set 2	
	Hazard ratio [95%CI]	P-value	Hazard ratio [95%CI]	P-value
**Pathologist Grade Group**				
1	* N/A		1.0 (reference)	-
2			3.85 [1.39, 10.70]	p=0.010
3			4.68 [1.49, 14.76]	p=0.009
4			14.30 [5.03, 40.62]	p<0.001
5			35.87 [13.00, 98.97]	p<0.001
**A.I. risk group**				
1	1.0 (reference)	-	1.0 (reference)	-
2	2.83 [1.34, 5.98]	p=0.006	0.71 [0.17, 2.97]	p=0.641
3	9.55 [4.70, 19.37]	p<0.001	6.23 [2.19, 17.69]	p<0.001
4	13.99 [6.77, 28.92]	p<0.001	13.16 [4.74, 36.54]	p<0.001
5	39.96 [20.04, 79.69]	p<0.001	35.54 [13.26, 95.27]	p<0.001

*Not available because pathologist Grade Groups were not available for all cases in validation set 1 due to the earlier time period.

Supplementary Table S7. Multivariable hazard ratios for A.I. Gleason pattern percentages

	Validation Set 1 (All cases from 1995-2014) n=2807 cases		Validation Set 2 (Cases from 2000-2014 with a pathologist GG) n=1517 cases		Cases in Validation Set 1 but not in Validation Set 2 n=1290 cases	
**Covariate**	**HR**	**p**	**HR**	**p**	**HR**	**p**
**A.I. Gleason pattern percentage**						
%GP3	1.00 (reference)	n/a	1.00 (reference)	n/a	1.00 (reference)	n/a
%GP4	1.34 [1.19, 1.50]	p<0.001	1.35 [1.17, 1.57]	p<0.001	1.30 [1.07, 1.58]	p=0.009
%GP5	1.54 [1.41, 1.68]	p<0.001	1.53 [1.37, 1.70]	p<0.001	1.60 [1.38, 1.86]	p<0.001
**Pathology T category**						
T2	1.00 (reference)	n/a	1.00 (reference)	n/a	1.00 (reference)	n/a
T3-T4	5.55 [2.87, 10.74]	p<0.001	7.03 [3.28, 15.05]	p<0.001	2.85 [0.66, 12.38]	p=0.162
Unknown	3.16 [1.32, 7.55]	p=0.010	2.36 [0.28, 19.82]	p=0.429	3.09 [0.73, 13.14]	p=0.127
**Margin Status**						
Negative	1.00 (reference)	n/a	1.00 (reference)	n/a	1.00 (reference)	n/a
Positive	3.25 [1.07, 9.84]	p=0.037	1.69 [0.44, 6.48]	p=0.442	10.39 [1.18, 91.17]	p=0.035
Unknown	2.70 [0.96, 7.62]	p=0.060	1.81 [0.55, 5.94]	p=0.327	3.75 [0.43, 32.79]	p=0.232
**Pathology N category**						
N0	1.00 (reference)	n/a	1.00 (reference)	n/a	1.00 (reference)	n/a
N1-N3	1.69 [0.80, 3.56]	p=0.166	1.74 [0.73, 4.12]	p=0.211	2.08 [0.43, 10.04]	p=0.360
Unknown	1.04 [0.60, 1.79]	p=0.900	0.94 [0.50, 1.77]	p=0.848	1.32 [0.39, 4.47]	p=0.659

Supplementary Table S8. Multivariable C-Index for pathologist and A.I. grading

C-indices provided multivariable Cox regression models that include both a Gleason feature (e.g. pathologist Grade Groups) and a set of clinicopathologic variables: pathologic T-category, margin status, pathologic N-category. Cox regression was fit with an L2 penalty of 0.02 to assist with numerical convergence

	C-index [95%CI]	
	Validation Set 1	Validation Set 2
(A) Pathologist Grade Groups	N/A*	0.83 [0.76-0.89]
(B) A.I. risk score (continuous)	0.88 [0.84- 0.91]	0.89 [0.84- 0.93]
(C) A.I. risk groups (discretized)	0.87 [0.84- 0.91]	0.90 [0.86- 0.93]
(D) Average of (A) and (C)	N/A*	0.89 [0.84-0.93]

*Not available because pathologist Grade Groups were not available for all cases in validation set 1 due to the earlier time period.

Supplementary Table S9. 10-year disease-specific survival rates for disagreements between pathologist and A.I.

Kaplan-Meier estimates of 10-year survival rates for validation set 2 cases. For each pathologist Grade Group (GG) survival rates are shown for all cases (“All”), cases where the A.I. risk group was lower than the pathologist GG (“Lower”), cases where the A.I. risk group was the same as the pathologist GG (“Same”) and cases where the A.I. risk group was higher than the pathologist GG (“Higher”). Numbers in square braces indicate 95% confidence intervals, with n indicating the size of the group.

Pathologist Grade Group	All	A.I. risk group		
		Lower	Same	Higher
**Grade Group 1**	1.00 [0.99, 1.00] n=608	N/A	1.00 [1.00, 1.00] n=327	0.99 [0.97, 1.00] n=281
**Grade Group 2**	0.98 [0.96, 0.99] n=473	1.00 [1.00, 1.00] n=212	0.98 [0.94, 1.00] n=179	0.93 [0.84, 0.97] n=82
**Grade Group 3**	0.99 [0.95, 1.00] n=224	1.00 [1.00, 1.00] n=117	0.98 [0.87, 1.00] n=56	0.97 [0.81, 1.00] n=51
**Grade Group 4**	0.92 [0.83, 0.96] n=127	1.00 [1.00, 1.00] n=83	0.81 [0.56, 0.93] n=26	0.72 [0.33, 0.91] n=18
**Grade Group 5**	0.83 [0.70, 0.90] n=85	0.93 [0.74, 0.98] n=53	0.65 [0.42, 0.81] n=32	N/A

Supplementary Table S10. Assessment of intra-scanner and inter-scanner variability on Gleason pattern quantitations across 144 scans. 12 high-grade specimens were scanned using 4 digital pathology scanners: Leica AT2, Philips UFS, 3D Histech P250, and Hamamatsu Nanozoomer, each 3 times. We computed the intra-scanner and inter-scanner correlation (as measured by R^2) for %GP4 and %GP5 values, which span a large range (0-100). Median R^2 across comparisons are presented. Ranges across comparisons are presented in parentheses.

A) Intra-scanner R^2

	Leica AT2	Philips UFS	3D Histech P250	Hamamatsu Nanozoomer
%GP4	0.997 (0.996-0.999)	0.999 (0.999-1.000)	0.999 (0.999-1.000)	0.998 (0.998-0.999)
%GP5	0.998 (0.998-0.999)	1.000 (0.999-1.000)	0.999 (0.999-1.000)	0.999 (0.999-0.999)

B) Inter-scanner R^2 for %GP4 (3 scan settings per scanner).

	Leica AT2	Philips UFS	3D Histech P250	Hamamatsu Nanozoomer
Leica AT2	1			
Philips UFS	0.977 (0.976-0.978)	1		
3D Histech P250	0.992 (0.991-0.994)	0.974 (0.970-0.977)	1	
Hamamatsu Nanozoomer	0.976 (0.972-0.979)	0.956 (0.954-0.957)	0.965 (0.959-0.970)	1

C) Inter-scanner R^2 for %GP5 (3 scan settings per scanner)

	Leica AT2	Philips UFS	3D Histech P250	Hamamatsu Nanozoomer
Leica AT2	1			
Philips UFS	0.977 (0.976-0.978)	1		
3D Histech P250	0.992 (0.991-0.994)	0.949 (0.948-0.951)	1	
Hamamatsu Nanozoomer	0.976 (0.972-0.979)	0.944 (0.942-0.945)	0.974 (0.970-0.977)	1

Supplementary Figures

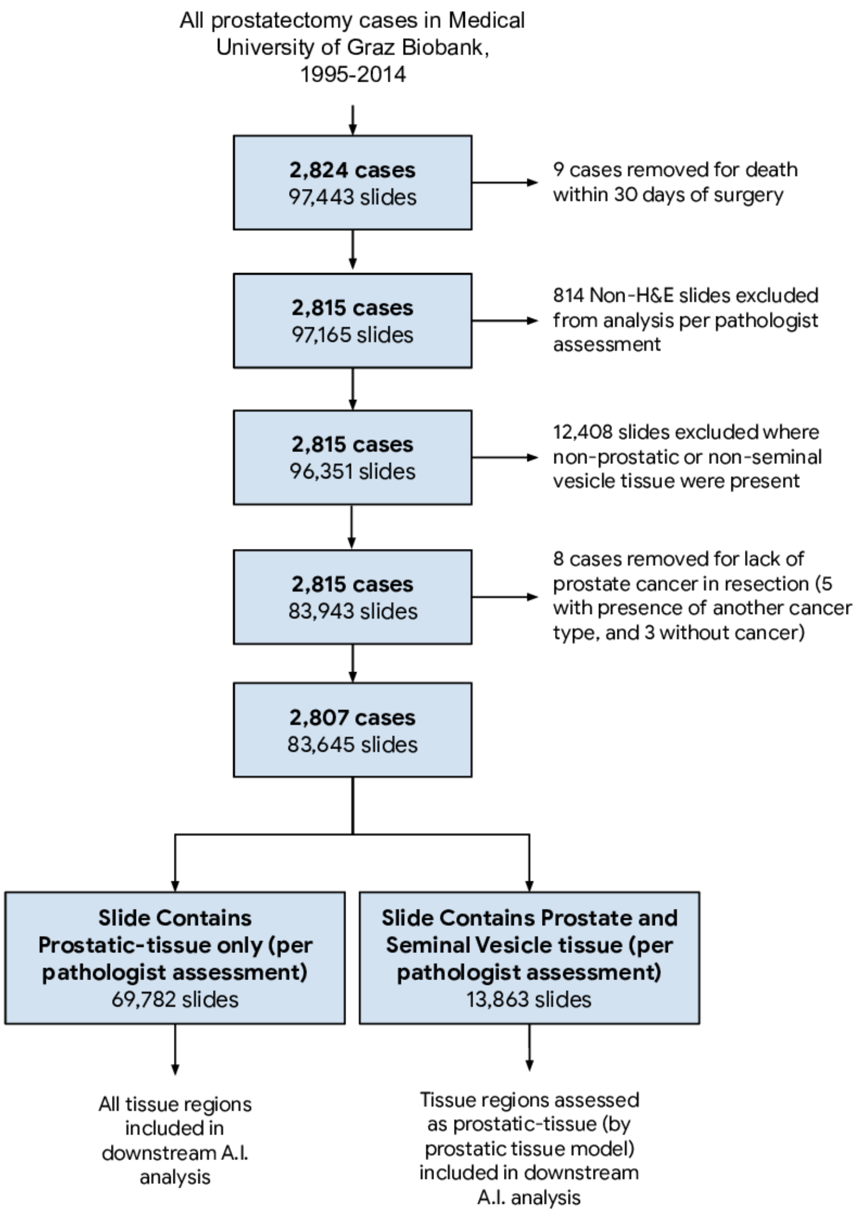

Supplementary Figure S1. STARD diagram of inclusion/exclusion criteria.

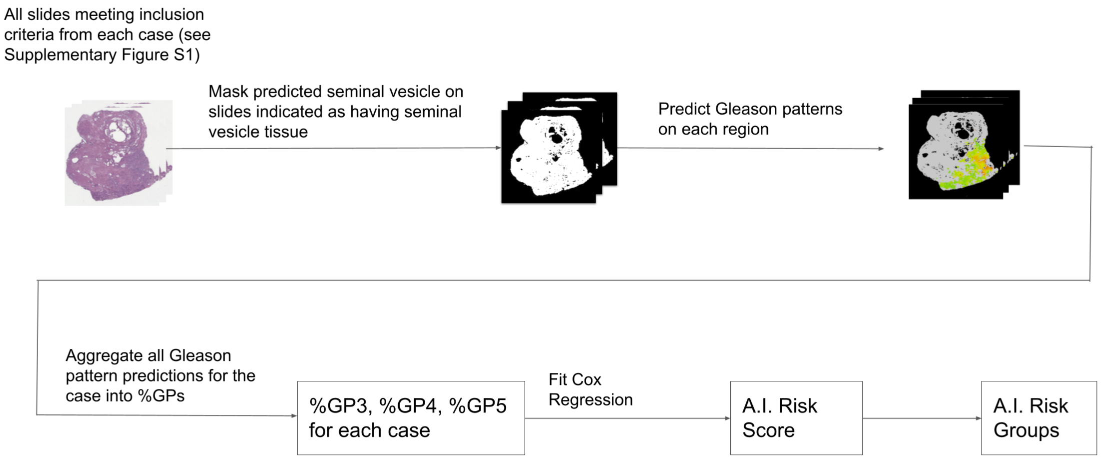

Supplementary Figure S2. Workflow diagram for A.I. risk scoring.

(A) C-indices for A.I. and Pathologist risk assessment.

		A.I. Risk Score	Pathologist	Delta
Validation Set 1	All cases (n=2817)	0.84 [0.80-0.87]	N/A	N/A
	Cases without treatment (n=2509)	0.83 [0.79, 0.86]	N/A	N/A
	Cases with treatment (n=298)	0.80 [0.66, 0.93]	N/A	N/A
Validation Set 2	All cases (n=1517)	0.87 [0.80, 0.92]	0.79 [0.71, 0.85]	0.08 [0.01, 0.16]
	Cases without treatment (n=1327)	0.85 [0.78, 0.91]	0.77 [0.69, 0.85]	0.08 [0.01, 0.17]
	Cases with treatment (n=190)	0.88 [0.79, 0.97]	0.79 [0.59, 0.92]	0.09 [-0.03, 0.24]

(B) KM Curve for cases without known additional treatment, Validation Set 1

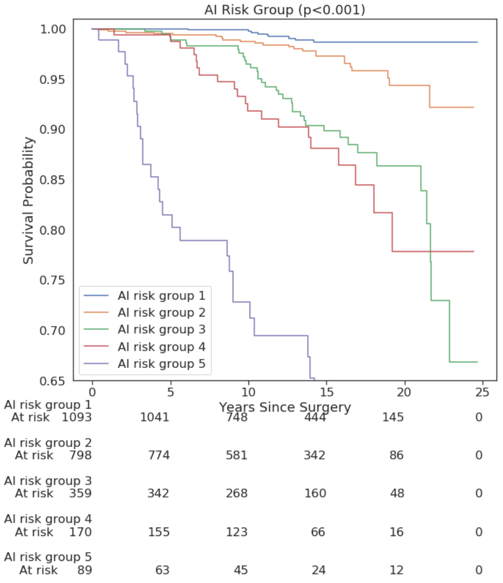

(C) KM curve for cases without known additional treatment, Validation Set 2

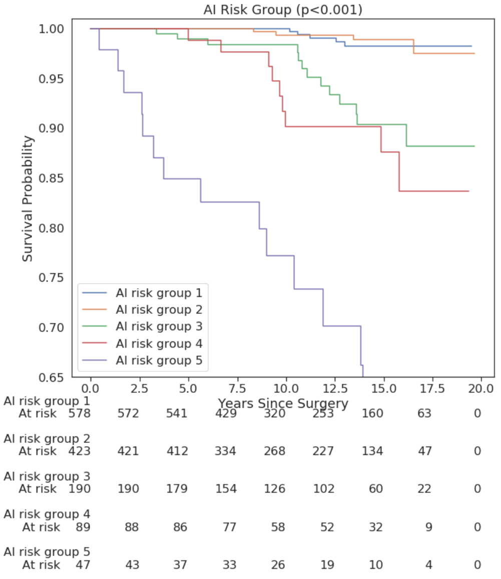

Supplementary Figure S3. C-indices and Kaplan-Meier curves for A.I. and pathologist risk stratification of patients, including and excluding cases with known neo-adjuvant, adjuvant, or salvage therapy. (A) C-indices for Pathologist and AI grading. 95%CI are presented in brackets. (B) Kaplan-Meier curves for AI grading across cases in Validation Set 1 excluding those that received known adjuvant or salvage therapy. (C) Kaplan-Meier curves for AI grading across cases in Validation Set 2 excluding those that received known adjuvant or salvage therapy.

(A) A.I. Risk Groups within Pathologic T-Stage 1-2 Cases

(B) A.I. Risk Groups within Pathologic T-Stage 3-4 Cases:

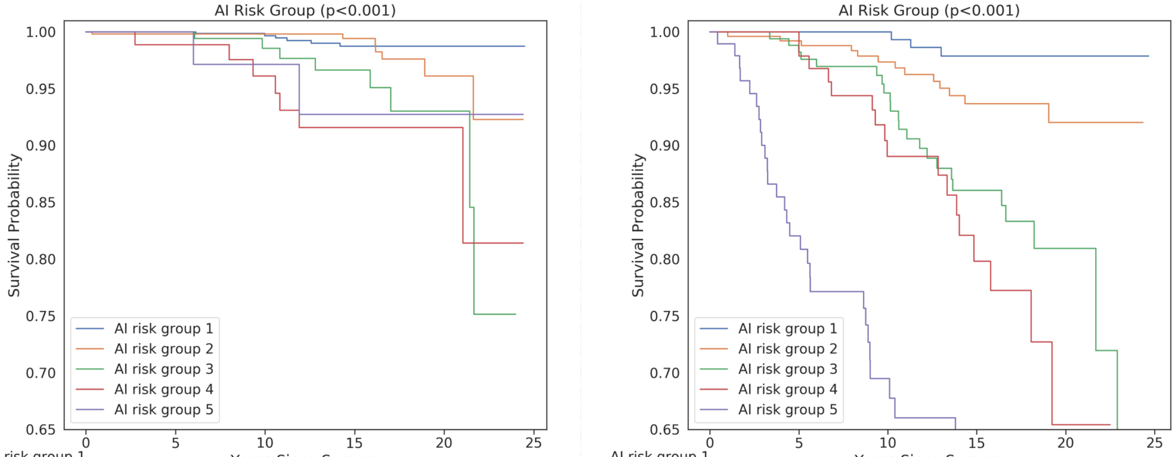

(A)

	0	5	10	15	20	25
AI risk group 1 At risk	813	770	505	312	86	0
AI risk group 2 At risk	502	488	338	213	42	0
AI risk group 3 At risk	192	182	115	71	19	0
AI risk group 4 At risk	90	86	65	42	14	0
AI risk group 5 At risk	43	38	26	15	5	0

(B)

	0	5	10	15	20	25
AI risk group 1 At risk	192	181	148	116	55	0
AI risk group 2 At risk	256	244	183	121	45	0
AI risk group 3 At risk	172	161	120	77	22	0
AI risk group 4 At risk	100	93	62	35	8	0
AI risk group 5 At risk	96	70	40	20	9	0

Supplementary Figure S4. Kaplan-Meier curves for A.I. risk groups by T-category

Kaplan-Meier curves for A.I. risk groups within pathologic T-stage categories of A) 1-2 and B) 3-4 for validation set 1.

References

1. Website. https://www.cancer.gov/tcga.
2. Nagpal, K. *et al.* Development and validation of a deep learning algorithm for improving Gleason scoring of prostate cancer. *NPJ Digit Med* **2**, 48 (2019).
3. Nagpal, K. *et al.* Development and Validation of a Deep Learning Algorithm for Gleason Grading of Prostate Cancer From Biopsy Specimens. *JAMA Oncol* (2020) doi:10.1001/jamaoncol.2020.2485.
4. P., P. G. *et al. Protocol for the examination of specimens from patients with carcinoma of the prostate gland*. https://documents.cap.org/protocols/cp-malegenital-prostate-radicalprostatectomy-20-4101.pdf (2020).
