# Supplementary material for: Predicting prostate cancer specific-mortality with artificial intelligence-based Gleason grading
Source: Commun Med (Lond). 2021 Jun 30;1:10. doi: 10.1038/s43856-021-00005-3 (PMC9053226; doi:10.1038/s43856-021-00005-3)
Supplement: Supplementary file 2 — Reporting Summary [file 43856_2021_5_MOESM2_ESM.pdf]

nature research

Corresponding author(s): Yun Liu
Last updated by author(s): Apr 30, 2021

Reporting Summary

Nature Research wishes to improve the reproducibility of the work that we publish. This form provides structure for consistency and transparency in reporting. For further information on Nature Research policies, see our Editorial Policies and the Editorial Policy Checklist.

Statistics

For all statistical analyses, confirm that the following items are present in the figure legend, table legend, main text, or Methods section.

n/a	Confirmed	
☐	☒	The exact sample size (n) for each experimental group/condition, given as a discrete number and unit of measurement
☐	☒	A statement on whether measurements were taken from distinct samples or whether the same sample was measured repeatedly
☐	☒	The statistical test(s) used AND whether they are one- or two-sided<br>*Only common tests should be described solely by name; describe more complex techniques in the Methods section.*
☐	☒	A description of all covariates tested
☐	☒	A description of any assumptions or corrections, such as tests of normality and adjustment for multiple comparisons
☐	☒	A full description of the statistical parameters including central tendency (e.g. means) or other basic estimates (e.g. regression coefficient) AND variation (e.g. standard deviation) or associated estimates of uncertainty (e.g. confidence intervals)
☐	☒	For null hypothesis testing, the test statistic (e.g. F, t, r) with confidence intervals, effect sizes, degrees of freedom and P value noted<br>*Give P values as exact values whenever suitable.*
☒	☐	For Bayesian analysis, information on the choice of priors and Markov chain Monte Carlo settings
☒	☐	For hierarchical and complex designs, identification of the appropriate level for tests and full reporting of outcomes
☐	☒	Estimates of effect sizes (e.g. Cohen's d, Pearson's r), indicating how they were calculated

Our web collection on statistics for biologists contains articles on many of the points above.

Software and code

Policy information about availability of computer code

Data collection	All available slides for archived prostate cancer resection cases between 1995 and 2014 in the BioBank Graz at the Medical University of Graz were retrieved, de-identified, and scanned using a Leica Aperio AT2 scanner at 40X magnification (0.25 µm/pixel). Primary and secondary Gleason patterns (Gleason Scores) were extracted from the original pathology reports, along with pathologic TNM staging, and patient age at diagnosis. Disease-specific survival (DSS) was inferred from International Classification of Diseases (ICD) codes from the Statistik Austria database. Codes considered for prostate-cancer related death were C61 and C68.
Data analysis	The open-sourced library TensorFlow was used to develop the models, and the lifelines library and custom Python code was used for statistical analysis. The trained model has not yet undergone regulatory review and cannot be made available at this time. Interested researchers can contact C.M. (cmermel@google.com) for questions on its status and access.

For manuscripts utilizing custom algorithms or software that are central to the research but not yet described in published literature, software must be made available to editors and reviewers. We strongly encourage code deposition in a community repository (e.g. GitHub). See the Nature Research guidelines for submitting code & software for further information.

Data

Policy information about availability of data

All manuscripts must include a data availability statement. This statement should provide the following information, where applicable:

- Accession codes, unique identifiers, or web links for publicly available datasets
- A list of figures that have associated raw data
- A description of any restrictions on data availability

This study analyzed datasets containing archived anonymized pathology slides, clinicopathologic variables, and outcomes information from the Institute of

Pathology and the Biobank at the Medical University of Graz. The datasets are not publicly available to respect patient privacy, and interested researchers should contact K.Z. (kurt.zatloukal@medunigraz.at) to inquire about access; requests for non-commercial academic use will be considered and require ethics review.

Field-specific reporting

Please select the one below that is the best fit for your research. If you are not sure, read the appropriate sections before making your selection.

☒ Life sciences ☐ Behavioural & social sciences ☐ Ecological, evolutionary & environmental sciences

For a reference copy of the document with all sections, see nature.com/documents/nr-reporting-summary-flat.pdf

Life sciences study design

All studies must disclose on these points even when the disclosure is negative.

Sample size	All available cases were used. Statistical power was estimated based on a range of possible effect sizes, a single primary analysis was pre-specified because estimated power would not enable multiple testing correction.
Data exclusions	Cases with deaths within 30 days of surgery were excluded. Immunohistochemically stained slides were excluded from analysis and only slides containing primarily prostatic tissue were included. Slides containing exclusively prostatic tissue were included in their entirety. Slides with both prostatic tissue and seminal vesicle tissue were included, but processed using a prostatic tissue model meant to provide only prostatic tissue to the Gleason grading model. Please see Supplementary Figure 1 for more details.
Replication	This is the "replication" (validation) study of an exploratory survival analysis from a prior paper, Nagpal et al. npj Digital Medicine 2019.
Randomization	N/A.
Blinding	All grading or labeling were done blinded to model predictions.

Reporting for specific materials, systems and methods

We require information from authors about some types of materials, experimental systems and methods used in many studies. Here, indicate whether each material, system or method listed is relevant to your study. If you are not sure if a list item applies to your research, read the appropriate section before selecting a response.

Materials & experimental systems

n/a	Involved in the study	
☒	☐	Antibodies
☒	☐	Eukaryotic cell lines
☒	☐	Palaeontology and archaeology
☒	☐	Animals and other organisms
☒	☐	Human research participants
☐	☒	Clinical data
☒	☐	Dual use research of concern

Methods

n/a	Involved in the study	
☒	☐	ChIP-seq
☒	☐	Flow cytometry
☒	☐	MRI-based neuroimaging

Clinical data

Policy information about clinical studies

All manuscripts should comply with the ICMJE guidelines for publication of clinical research and a completed CONSORT checklist must be included with all submissions.

Clinical trial registration	N/A
Study protocol	N/A
Data collection	Retrospective analysis of de-identified data, please see data collection above.
Outcomes	Disease-specific survival (DSS) was inferred from International Classification of Diseases (ICD) codes from the Statistik Austria database. Codes considered for prostate-cancer related death were C61 and C68.
